# Action- and Language-Conditioned Video Assessment for Embodied Control

**DOI:** 10.3390/s26165046

**Published:** 2026-08-08

**Authors:** Hwanhee Kim, Jaehyun Jang, Seungmin Cha, Hyeonseo Yun, Donghoon Lee, Chang D. Yoo

**Affiliations:** 1 Robotics Program, Korea Advanced Institute of Science and Technology (KAIST), Daejeon 34141, Republic of Korea; khhandrea@kaist.ac.kr; 2 School of Electrical Engineering, Korea Advanced Institute of Science and Technology (KAIST), Daejeon 34141, Republic of Korea; jhjangjh@kaist.ac.kr (J.J.); cseungmin7@kaist.ac.kr (S.C.); hyunseo@kaist.ac.kr (H.Y.); dh_lee99@kaist.ac.kr (D.L.)

**Keywords:** vision-language models, video assessment, trajectory evaluation, embodied control, task-progress feedback, embodied AI

## Abstract

Vision-based embodied agents executing multi-step natural language instructions require feedback mechanisms that assess task progress over complete trajectories. Conventional approaches based on final-frame matching or continuous embedding similarity may overlook intermediate transitions that are necessary for determining whether an instruction has been completed. We propose ALVA (Action- and Language-Conditioned Video Assessment), a trajectory evaluator that conditions its assessment on visual observations, the executed action sequence, and the natural language instruction. The method uses a pre-trained vision-language model (VLM) in two stages: it first summarizes frame-to-frame visual transitions conditioned on the executed actions and then assesses the generated summary with respect to the instruction to produce a discrete trajectory-level progress score. In simulated 3D household environments, ALVA exhibits a conservative assessment pattern with near-zero false-positive rates. When used as terminal feedback for closed-loop policy optimization, it provides more effective feedback than the evaluated static image and embedding-based visual baselines and reduces the performance gap to a ground-truth oracle. These results support action- and language-conditioned video assessment as an interpretable feedback mechanism for the evaluated simulated embodied-control tasks.

## 1. Introduction

Embodied agents increasingly rely on visual observations to execute sequential actions in complex environments. For multi-step tasks specified by natural language, reliable policy optimization requires an evaluator that can assess how a trajectory progresses toward the instruction rather than considering only an isolated final observation [1,2,3].

A common zero-shot strategy compares a final visual observation with a language instruction using pre-trained vision-language representations [4,5,6]. This approach is simple, but it can discard intermediate task progress and cannot directly indicate which executed actions correspond to the observed visual changes. Video-language and robotic representation learning methods incorporate temporal information [7,8,9], yet many still derive feedback from embedding similarity rather than an explicit interpretation of action-conditioned transitions.

We therefore formulate trajectory assessment as an action- and language-conditioned video evaluation problem. The evaluator receives a video trajectory, the executed action sequence, and a natural language instruction, and determines whether the observed transitions indicate meaningful task progress. This formulation focuses on action-to-transition correspondence and task-progress assessment.

To address this problem, we propose ALVA (Action- and Language-Conditioned Video Assessment). The system represents each interaction as a video trajectory composed of visual observations, executed control commands, and a natural language instruction. A pre-trained vision-language model [10,11,12] is used as a trajectory-level evaluator through a two-stage querying process. In Stage 1, the model performs action-conditioned transition summarization by describing frame-to-frame visual changes in relation to the executed action sequence. In Stage 2, the generated summary is assessed with respect to the language instruction to estimate task progress. The resulting discrete score provides trajectory-level feedback without relying only on static final-frame matching.

We evaluate ALVA in simulated 3D household environments based on ALFRED and AI2-THOR, where agents execute sequential actions to complete natural language instructions [3,13]. The results show that ALVA provides more effective feedback than the evaluated static image-text and embedding-based visual baselines [4,7,9]. The findings are limited to the tested simulated household tasks and VLM backbones.

The main contributions of this study are as follows:

- We propose ALVA, an action- and language-conditioned video assessment framework that evaluates trajectories from visual observations, executed control commands, and natural language instructions.
- We introduce a two-stage vision-language querying process that performs action-conditioned transition summarization followed by language-conditioned task-progress assessment.
- We formulate the resulting progress evaluation as an interpretable trajectory-level feedback signal that can support downstream autonomous control modules, including online off-policy policy optimization.
- We empirically validate the proposed system in complex 3D embodied environments and demonstrate that it provides more effective feedback than single-frame and embedding-similarity-based evaluation methods.

The remainder of this paper is organized as follows: Section 2 reviews related work. Section 3 introduces the preliminary formulations. Section 4 details the proposed ALVA methodology. Section 5 describes the experimental setup, and Section 6 presents the results and analysis. Finally, Section 7 and Section 8 provide the discussion and conclusions, respectively.

## 2. Related Work

### 2.1. Video Understanding for Embodied Control

Autonomous control systems rely on continuous sensor observations to perceive the environment, monitor state changes, and execute sequential decisions. In vision-based systems, these observations are naturally represented as video streams rather than isolated images. Video understanding is therefore important in embodied navigation and robotic manipulation, where the system must reason about temporal changes in the scene. Unlike static image recognition, video interpretation requires identifying object motion, interaction events, and state transitions over time. This is particularly important for autonomous control, where visual changes are often induced by the system’s own actions.

Recent advances in vision-language models have enabled strong image-text and video-text alignment capabilities. Models such as CLIP provide general-purpose image-text representations that can be used for zero-shot recognition and task specification [4]. More recent large vision-language models further extend multimodal reasoning and instruction-following capabilities across visual and textual inputs [10,11,12,14,15,16]. However, image-level alignment alone is often insufficient for evaluating sequential control behavior, because task progress may depend on intermediate transitions that are not visible in a single frame. Video-language models and visual representation learning methods, including R3M, LIV, and RoboCLIP, incorporate temporal or task-conditioned visual information and have shown promise for interpreting robot trajectories and visual demonstrations [7,8,9,17]. Nevertheless, many existing methods still evaluate trajectories through embedding similarity or task-specific descriptions, which can limit their ability to explicitly relate observed visual changes to the executed action sequence.

### 2.2. Language-Guided Autonomous Control

Language-guided autonomous control aims to enable agents to execute tasks specified by natural language instructions. This problem has been widely studied in language-conditioned reinforcement learning and embodied instruction following, where an agent receives a language instruction and produces a sequence of actions to complete the task [1,2,18,19]. Benchmarks such as ALFRED and CALVIN provide visually grounded environments with natural language instructions and sequential interaction requirements, making them suitable for evaluating embodied agents in complex 3D scenes [3,13,20]. Related work has also studied grounded navigation, manipulation, and language-conditioned behavior learning in simulated and real environments [21,22,23,24,25].

A major challenge in language-guided control is task evaluation. In many environments, evaluation rules require access to privileged state information, manually engineered task logic, or demonstrations that specify desired behavior. For example, household manipulation tasks may require checking whether an object has been picked up, moved, cleaned, cooled, or placed in a target receptacle. Such task-specific evaluation functions can be difficult to scale across diverse instructions and environments. Prior work has explored demonstrations, inverse reinforcement learning, and language-conditioned reward modeling to reduce manual specification effort [26,27,28,29,30]. However, these approaches often require substantial human data or task-specific supervision. In contrast, our work focuses on generating trajectory-level task assessments directly from visual observations, executed control commands, and natural language instructions.

### 2.3. Foundation Models for Task Evaluation

Foundation models have increasingly been explored as task evaluators for embodied systems. Large language models can generate task specifications or executable reward programs from natural-language descriptions [31,32,33,34,35]. Methods such as Language to Rewards, Text2Reward, and Eureka operate through programmatic interfaces and generally construct reward code using simulator variables, object attributes, environment APIs, or other structured task information. Their output is an executable reward function rather than a direct assessment of a recorded visual trajectory.

Vision-language models provide a different interface by evaluating observations directly. Existing approaches use image-text or video-text similarity as a proxy for task completion [5,6,8,9,36], while more recent work uses large VLMs to provide success judgments or task-level feedback [37,38]. Human-feedback research has separately studied preference-based and rating-based supervision [39,40,41,42].

ALVA addresses the observation-based assessment setting. It receives visual observations, the executed action sequence, and a natural-language instruction, and returns a trajectory-level task-progress score without accessing privileged simulator states. Programmatic reward-generation methods are therefore discussed as related alternatives but are not included as direct baselines because they use a different evaluation interface. Our empirical comparisons are limited to the evaluated zero-shot visual and video-language baselines that can be integrated into the same observation-based control pipeline.

### 2.4. Action- and Language-Conditioned Video Assessment

Trajectory evaluation for embodied control should account for both the executed action sequence and the task instruction. A video trajectory is not merely a collection of frames: the actions provide temporal context for interpreting the observed transitions, while the instruction defines which transitions constitute task-relevant progress. We therefore describe the target problem as action- and language-conditioned video assessment.

Prior video reasoning research shows that models can struggle to interpret temporal relationships and action-dependent events beyond static scene recognition [43,44,45,46]. These studies are primarily formulated as video question answering or event-structure analysis, whereas the present work focuses on assigning task-progress feedback to embodied-control trajectories.

ALVA addresses this trajectory-assessment problem through two stages: action-conditioned transition summarization and language-conditioned task-progress assessment. Static image-text matching can indicate whether a final frame resembles a target description, while embedding-based video similarity can capture temporal patterns. In contrast, the proposed decomposition produces an explicit summary of the action-to-transition correspondence before assigning a discrete task-progress score.

## 3. Preliminaries

### 3.1. Action-Conditioned Video Trajectories

Let $I_{t}\in I$ denote the RGB observation at time step *t*, $u_{t}\in U$ the executed control command, and l∈L the natural-language instruction. We represent an interaction of length *T* as an action-conditioned video trajectory

(1) $$\tau =(I_{0},u_{0},I_{1},u_{1},\ldots ,I_{T-1},u_{T-1},I_{T}).$$

The trajectory preserves the temporal order of the visual observations and the corresponding executed commands.

### 3.2. Action- and Language-Conditioned Task Assessment

We define the trajectory evaluator as

(2) F:T×L→R,

where R={0,1,…,k−1} is a discrete task-progress scale, and T is the space of such trajectories. In the experiments, k=4: a score of 0 denotes no task-relevant progress and a score of 3 denotes full task completion. The evaluator uses the visual transitions, executed commands, and instruction rather than relying only on the final observation.

### 3.3. Assessment-Driven Policy Optimization

The evaluator output is used as terminal feedback for downstream policy optimization. For a collected trajectory τ and instruction *l*, intermediate rewards are set to zero and the final reward is assigned as

(3) $$r_{T-1}=F\left(\tau ,l\right).$$

This formulation connects trajectory-level task-progress assessment to downstream control without requiring a manually engineered task-specific reward function. The policy-optimization algorithm itself is not a contribution of this work.

### 3.4. Vision-Language Models as Trajectory Evaluators

We instantiate *F* with a pre-trained vision-language model. The visual prompt constructor Ω maps sampled observations from τ into a composite visual input. The summarization prompt constructor $\Psi_{S}$ asks the model to describe action-conditioned visual transitions, and the task-progress assessment prompt constructor $\Psi_{C}$ asks it to assign a discrete score from the generated summary. Section 4.1 specifies this two-stage process.

## 4. Materials and Methods

ALVA instantiates the autonomous evaluator F(τ,l) defined in the Preliminaries through a structured two-stage VLM querying pipeline. Given an action-conditioned video trajectory τ and a language instruction *l*, the objective is to infer a trajectory-level feedback score r∈R directly from visual observations and executed control commands. The framework uses a pre-trained VLM *H* as the core trajectory evaluator, together with a visual prompt constructor Ω, a summarization prompt constructor $\Psi_{S}$, and a task-progress assessment prompt constructor $\Psi_{C}$. This design allows the system to approximate F(τ,l) without manually engineered task-specific evaluation rules.

**Figure 1:**
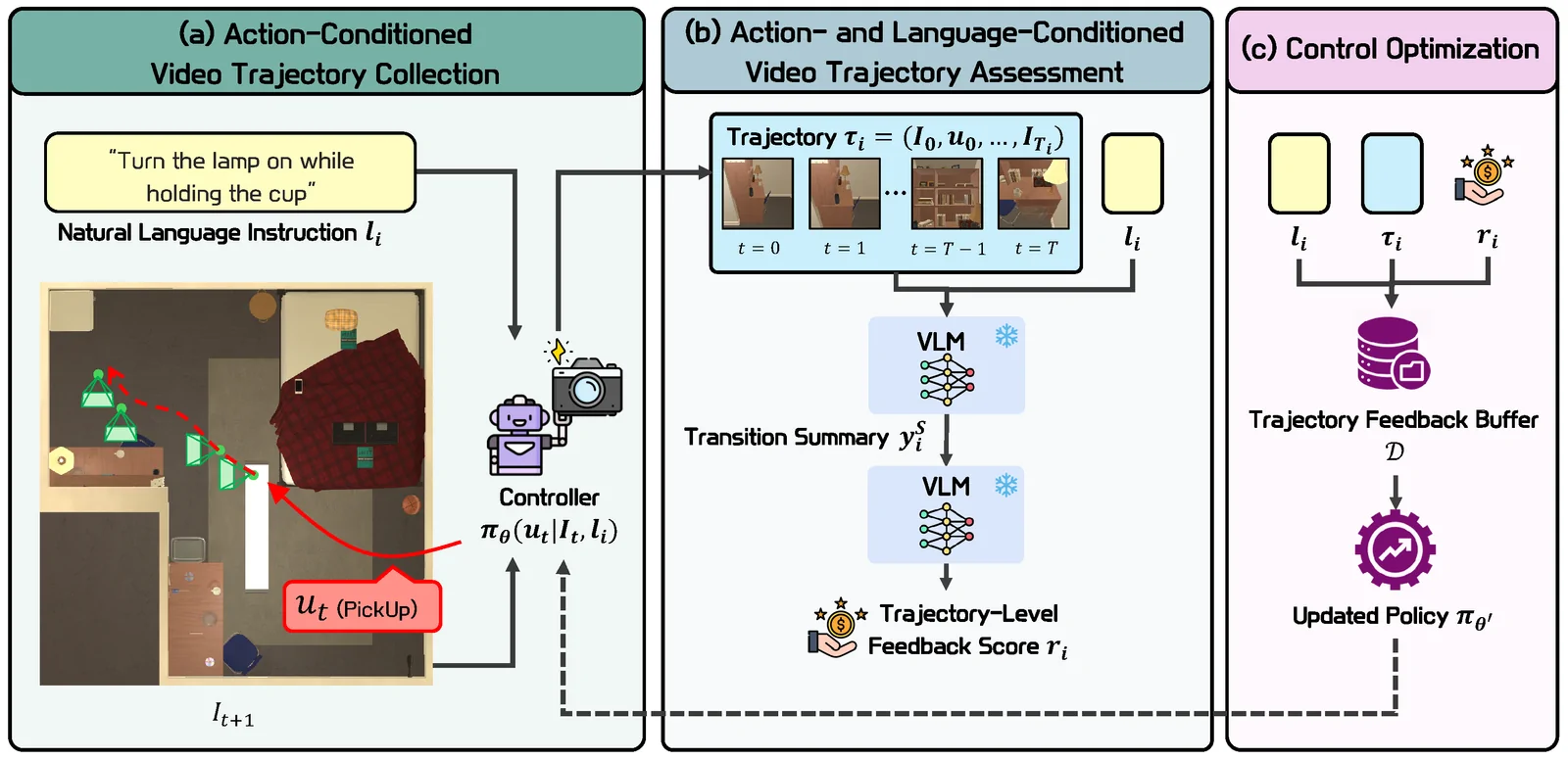
Overview of ALVA. (**a**) A language-conditioned controller collects an action-conditioned video by executing control commands from visual observations under a natural language instruction. (**b**) A two-stage VLM trajectory evaluator first summarizes action-induced visual transitions from the collected stream and then estimates task progress with respect to the instruction. The snowflake symbol indicates that the pre-trained VLM is frozen and its parameters are not updated during control optimization. (**c**) The resulting trajectory-level feedback score is stored in the evaluated trajectory buffer and used for assessment-driven control optimization.

The overall procedure is illustrated in Figure 1 and summarized in Algorithm 1. The controller first interacts with the environment to collect an action-conditioned video trajectory. The collected trajectory is then converted into a composite visual input and a structured text prompt. In the first stage, the VLM summarizes action-induced visual transitions. In the second stage, the generated summary is evaluated with respect to the instruction to produce a discrete task-progress score. The resulting score is directly used as a trajectory-level feedback signal for downstream autonomous control.
**Algorithm 1** ALVA closed-loop evaluation and optimization procedure.  1:**Input**: pre-trained VLM *H*, visual prompt constructor Ω, prompt constructors $\Psi_{S}$ and $\Psi_{C}$  2:**Initialize**: Language-conditioned controller $\pi_{\theta }$, trajectory buffer D  3:**while** not converged **do**  4:   Sample a language instruction $l_{i}∼L$  5:   Run $\pi_{\theta }$ to collect trajectory $\tau_{i}=\left(I_{0},u_{0},\ldots ,I_{T_{i}-1},u_{T_{i}-1},I_{T_{i}}\right)$  6:   Construct summarization inputs: $I_{i}^{\mathrm{vis}}=\Omega \left({\left\{I_{t}\right\}}_{t=0}^{T_{i}}\right)$, $p_{i}^{S}=\Psi_{S}\left({\left\{u_{t}\right\}}_{t=0}^{T_{i}-1},l_{i}\right)$  7:   Query for action-conditioned transition summary: $y_{i}^{S}=H\left(p_{i}^{S},I_{i}^{\mathrm{vis}}\right)$  8:   Construct assessment prompt: $p_{i}^{C}=\Psi_{C}\left(y_{i}^{S},l_{i}\right)$  9:   Query for trajectory-level feedback: $r_{i}=H\left(p_{i}^{C},\emptyset \right)$10:   Stored evaluated trajectory: $D\leftarrow D\cup \{\left(\tau_{i},l_{i},y_{i}^{S},r_{i}\right)\}$11:   Update $\pi_{\theta }$ using data sampled from D with an off-policy optimization algorithm12:**end while**

This procedure differs from approaches that first learn a separate feedback prediction model or evaluation model from human feedback or demonstrations [40,41,42]. Such intermediate modeling steps can introduce additional complexity and may be vulnerable to feedback misspecification or misgeneralization [47]. In contrast, ALVA queries the VLM directly as a trajectory-level evaluator and uses the resulting score as feedback. The central contribution is therefore not a new policy optimization algorithm, but an action- and language-conditioned video assessment mechanism that produces interpretable trajectory-level signals from visual observations and control histories.

### 4.1. Two-Stage Action- and Language-Conditioned Video Assessment

Natural language instructions in autonomous control scenarios can be highly compositional. For example, an instruction such as “Put the coffee cup in the sink, turn on the water, turn off the water, and pick up the coffee cup” involves multiple sequential sub-tasks. A reliable evaluator must therefore assess not only the final visual observation, but also the intermediate action-induced visual transitions that occur throughout the trajectory. This is particularly important because multiple valid control strategies may lead to the same task completion state, while failed trajectories can still resemble successful ones in the final frame.

**Figure 2:**
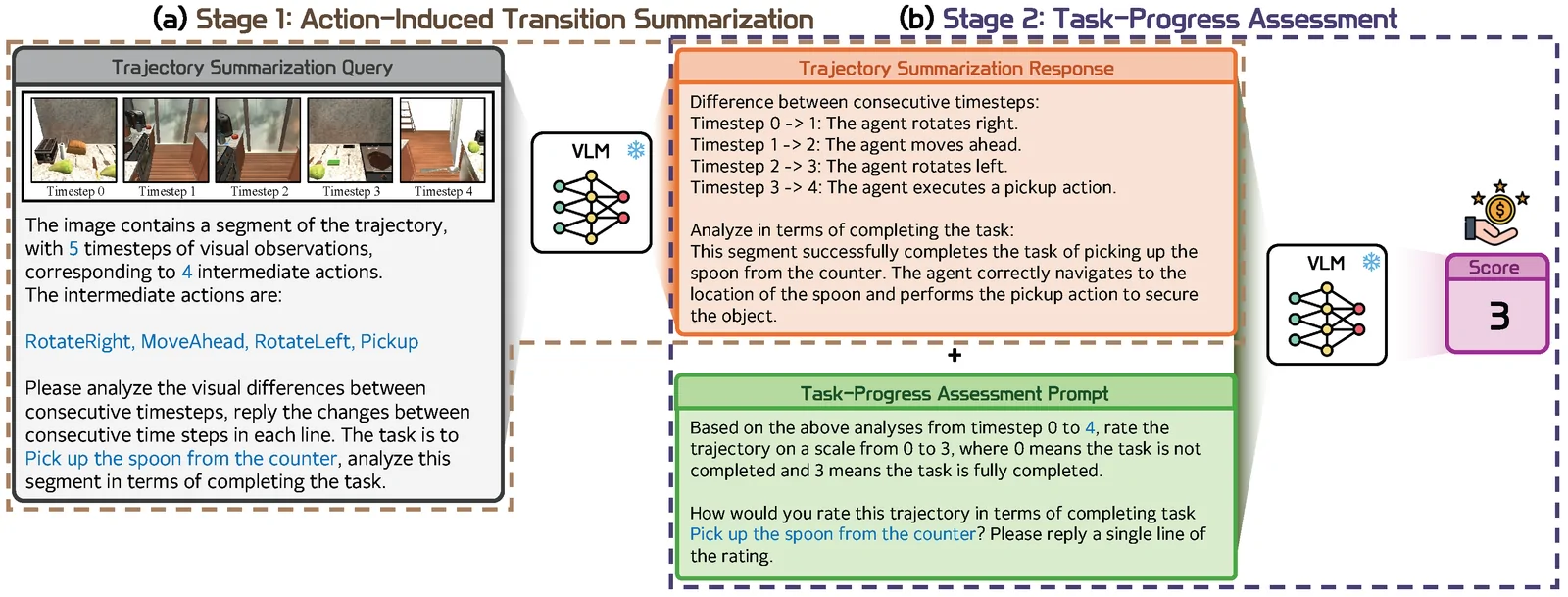
Illustration of the two-stage VLM querying process used in ALVA. Blue text indicates task- and trajectory-specific variables that are replaced according to the input. The snowflake symbol indicates that the pre-trained VLM is frozen and its parameters are not updated during control optimization. (**a**) In Stage 1, Action-Induced Transition Summarization, sampled visual observations are arranged into a composite visual prompt with timestep annotations, and the executed control commands are provided as textual context. The VLM then summarizes frame-to-frame visual transitions conditioned on the action sequence and the language instruction. (**b**) In Stage 2, Task-Progress Assessment, the generated transition summary is combined with a rating prompt and evaluated with respect to the original instruction. The VLM outputs a discrete trajectory-level feedback score, where higher scores indicate greater task progress toward full completion.

To address this issue, ALVA evaluates the full action-conditioned video trajectory rather than a single observation. As illustrated in Figure 2, the two-stage querying process separates transition interpretation from task-progress assessment. The first stage asks the VLM to summarize how the visual scene changes between consecutive observations, conditioned on the executed control commands and the language instruction. The second stage then uses this generated summary to infer whether the observed visual transitions constitute meaningful progress toward completing the instruction. This decomposition makes the assessment more interpretable and reduces the burden of asking the VLM to directly infer task completion from raw visual inputs alone.

Concretely, given an action-conditioned video trajectory $\tau_{i}$, the visual prompt constructor Ω maps the sampled visual observations into a composite visual input:

(4) $$I_{i}^{\mathrm{vis}}=\Omega \left({\left\{I_{t}\right\}}_{t=0}^{T_{i}}\right).$$

The composite input is constructed by concatenating sampled video frames and adding timestep annotations to preserve the temporal order. Such visual prompting strategies are related to prior studies showing that structured visual prompts can improve the reasoning behavior of pre-trained vision-language models [48,49,50].

The summarization prompt constructor $\Psi_{S}$ maps the executed control commands and the instruction into a text prompt:

(5) $$p_{i}^{S}=\Psi_{S}\left({\left\{u_{t}\right\}}_{t=0}^{T_{i}-1},l_{i}\right).$$

This prompt includes the sequence length, the executed command list, and an instruction asking the VLM to describe frame-to-frame visual changes in relation to the task. The VLM then generates an action-conditioned trajectory summary:

(6) $$y_{i}^{S}=H\left(p_{i}^{S},I_{i}^{\mathrm{vis}}\right).$$

The summary $y_{i}^{S}$ serves as an interpretable intermediate representation that describes the action-to-visual transitions observed in the trajectory.

For the second stage, the task-progress assessment prompt constructor $\Psi_{C}$ takes the generated summary and the original instruction as input:

(7) $$p_{i}^{C}=\Psi_{C}\left(y_{i}^{S},l_{i}\right).$$

The VLM is then queried to produce a discrete trajectory-level feedback score:

(8) $$r_{i}=H\left(p_{i}^{C},\emptyset \right),\;r_{i}\in R=\left\{0,1,\ldots ,k-1\right\}.$$

In our experiments, we use k=4, corresponding to the progress scale {0,1,2,3}, where 0 denotes no task-relevant progress and 3 denotes full task completion. This score is used directly as the feedback signal for downstream autonomous control. Unlike comparative feedback, which only indicates relative preference between trajectory pairs, this evaluative score provides an absolute assessment of task progress, which is better suited for multi-step instruction-following scenarios [41,42].

### 4.2. Implementation Details

Action-conditioned video trajectories can contain up to 50 interaction steps in the evaluated environments. To avoid constructing an excessively large composite input, we divide long trajectories into temporal segments and summarize each segment separately. Unless otherwise specified, the segment length is 10 frames. The segment-level summaries are concatenated and passed to the second-stage task-progress assessment.

The assessment-stage input is textual because it contains the generated transition summary and the original instruction. We use the same VLM backbone for both stages for implementation consistency. The experiments use the model aliases reported in Section 5; each alias was held fixed within an evaluation run. Provider-default decoding settings were used.

A task-progress response is accepted only when the complete stripped response is exactly one integer in {0,1,2,3}. An empty response, a response without a valid integer, or a value outside this range is treated as an invalid evaluator output. When this occurs during closed-loop optimization, no evaluator-derived feedback is stored for that trajectory and the corresponding evaluator-driven update is skipped. The current study does not evaluate sensitivity to prompt paraphrases or repeated queries.

Detailed downstream policy-optimization settings, environment configurations, and task lists are provided in Appendix A and Appendix B.

## 5. Experiments

We conduct experiments to evaluate the effectiveness and reliability of the proposed ALVA framework in visually grounded 3D embodied environments. The experiments are conducted in four household workspaces: Kitchen, Bathroom, Living Room, and Bedroom, using environments based on the ALFRED benchmark [3] and the AI2-THOR simulator [13]. Each environment requires an autonomous agent to complete natural language instructions through sequential interaction with visual observations and executed control commands.

Our experimental design has two main objectives. First, we evaluate whether ALVA can reliably assess action-conditioned video trajectories by comparing its trajectory-level feedback scores with ground-truth task completion labels. Second, we examine whether the generated feedback can support downstream autonomous control when used in a closed-loop setting. This design allows us to analyze both the diagnostic reliability of the trajectory evaluator and its utility as a feedback mechanism for language-guided autonomous control.

### 5.1. Experimental Setup

**Figure 3:**
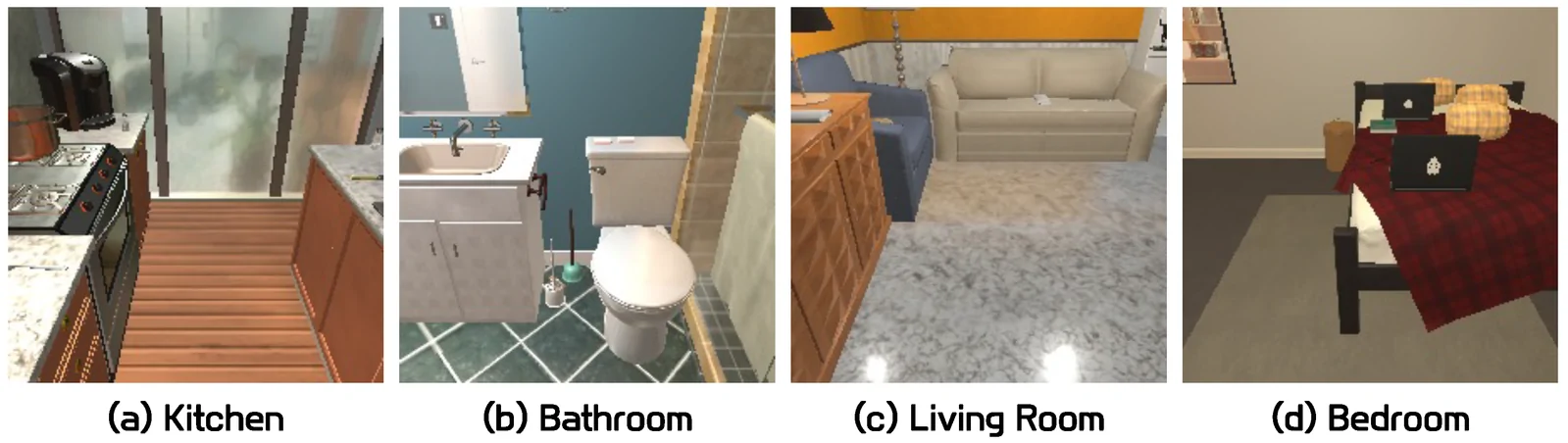
Representative egocentric views of the four unseen AI2-THOR floor plans used in the ALFRED-based experiments.

We evaluate ALVA on a set of visually grounded household tasks sampled from four ALFRED environments: Kitchen, Bathroom, Living Room, and Bedroom [3]. Each task consists of two sequential sub-tasks specified by natural language instructions. The agent receives RGB visual observations from an embodied camera and executes discrete navigation and interaction commands. An episode is considered successful only when both sub-tasks are completed. Representative egocentric views of the four evaluation environments are shown in Figure 3. Further details on the environment configuration, action space, and task instructions are provided in Appendix A and Appendix B.

For the VLM-based evaluator, we use the Gemini 1.5 Pro model alias as the primary vision-language model [11]. Given an action-conditioned video trajectory and a language instruction, ALVA generates a discrete trajectory-level feedback score r∈{0,1,2,3} through the two-stage querying process described in Section 4.1. A score of 0 indicates no task-relevant progress, while a score of 3 indicates full task completion. Unless otherwise specified, we use a temporal segment length of 10 frames during the first-stage video summarization process.

### 5.2. Baseline Evaluators

To evaluate the proposed action- and language-conditioned assessment framework, we compare ALVA against three zero-shot visual evaluation baselines—CLIP, R3M, and RoboCLIP—and include a ground-truth oracle as an upper-bound reference.

- CLIP [4]: A widely used vision-language foundational model. In our experiments, the CLIP-based evaluator computes the cosine similarity between the final visual observation $I_{T}$ and the natural-language instruction *l*. This serves as a standard static, single-frame image-text matching baseline that does not explicitly model temporal transitions.
- R3M [7]: A visual representation model pre-trained on the Ego4D human video dataset to capture robust temporal dynamics and human–object interactions. The R3M evaluator generates feedback by measuring the embedding similarity between the visual observations and the task context. While it incorporates visual information relevant to robotic control, it lacks the ability to explicitly summarize action-induced visual transitions.
- RoboCLIP [9]: A video-language representation model for embodied control. It generates a trajectory-level feedback score by computing the multimodal alignment score between the continuous video sequence τ and the language instruction *l*. Although it incorporates temporal visual information, the feedback is still derived from continuous embedding similarity rather than an explicit interpretation of action-to-visual transitions.
- Ground-Truth Oracle (Oracle): This upper-bound reference uses the environment-provided ground-truth task completion signal. It is not available in uninstrumented, open-ended autonomous systems and is used solely to contextualize the performance gap between the evaluation-driven policies and the environment-provided upper-bound reference.

To ensure a fair and isolated comparison, all baseline evaluators are integrated into the identical downstream closed-loop control pipeline with Implicit Q-learning (IQL) [51] without any human-engineered reward functions. The controller utilizes the similarity scores generated by these baselines directly as the optimization feedback, which is then evaluated against the discrete, action- and language-conditioned assessment signal *r* provided by ALVA.

### 5.3. Trajectory Data Collection

To analyze the assessment reliability of ALVA independently from the closed-loop optimization process, we first collect a dataset of action-conditioned video trajectories. Following prior work on ALFRED-based autonomous control [52], we deploy controllers at different optimization stages to generate approximately 500 trajectories per environment, yielding a mixture of successful, partially successful, and failed trajectories. For the confusion-matrix analysis, each trajectory is binarized using its ground-truth label: it is counted as positive only when both sub-tasks are completed, and negative otherwise.

Each trajectory is processed by ALVA to obtain a discrete feedback score r∈{0,1,2,3}. To compare the evaluator output with the binary ground-truth task completion label, we binarize the score as follows: r=3 is treated as a positive prediction, indicating full task completion, while r<3 is treated as a negative prediction. This binarization is used only for the diagnostic confusion-matrix analysis. Closed-loop optimization uses the original score in {0,1,2,3} directly as terminal feedback, so intermediate progress scores are not discarded.

### 5.4. Evaluation Metrics

We use standard classification metrics to evaluate the diagnostic reliability of trajectory-level task assessment. Let true positives denote successful trajectories correctly assigned the maximum feedback score, and false positives denote failed or incomplete trajectories incorrectly assigned the maximum score.

- Accuracy (Acc.): The proportion of correctly classified successful and unsuccessful trajectories among all evaluated trajectories.
- Precision (Prec.): The proportion of predicted successful trajectories that are actually successful. High precision indicates that the evaluator rarely assigns a false success signal to an incomplete trajectory.
- Recall (Rec.): The proportion of ground-truth successful trajectories correctly identified by the evaluator. High recall indicates that the evaluator detects most successful executions.
- F1-Score (F1): The harmonic mean of precision and recall, providing a balanced measure of evaluation reliability.

In the context of autonomous control, precision is particularly important because false positive feedback can reinforce incorrect behavior. However, recall is also relevant because an overly conservative evaluator may fail to reward successful trajectories. Therefore, we report all four metrics to characterize both the precision-recall trade-off and sensitivity of the proposed evaluation system.

### 5.5. Closed-Loop Feedback Evaluation

In addition to offline reliability analysis, we evaluate whether the trajectory-level feedback generated by ALVA can support downstream autonomous control. The generated feedback score is converted into the terminal reward signal for online off-policy optimization. We use IQL [51] as the downstream optimization algorithm, following prior work on ALFRED-like embodied environments [52]. The purpose of this experiment is not to propose a new policy optimization algorithm, but to test whether action- and language-conditioned video assessment produces feedback that is useful for improving autonomous task execution.

For each method, we train the controller using the corresponding feedback source and evaluate task completion rates over training. Performance is measured every fixed number of optimization iterations and averaged over multiple evaluation episodes. We report both learning curves and maximum task completion rates across the four environments.

## 6. Results

We evaluate the ALVA framework in three parts. Section 6.1, Section 6.2 and Section 6.3 analyze diagnostic reliability, temporal-window trade-offs, and VLM latency. Section 6.4 evaluates the use of the resulting scores as closed-loop terminal feedback. Section 6.5 examines the contributions of temporal annotations, executed actions, and evaluative scoring.

### 6.1. Analysis of Conservative Action-Conditioned Assessment

**Figure 4:**
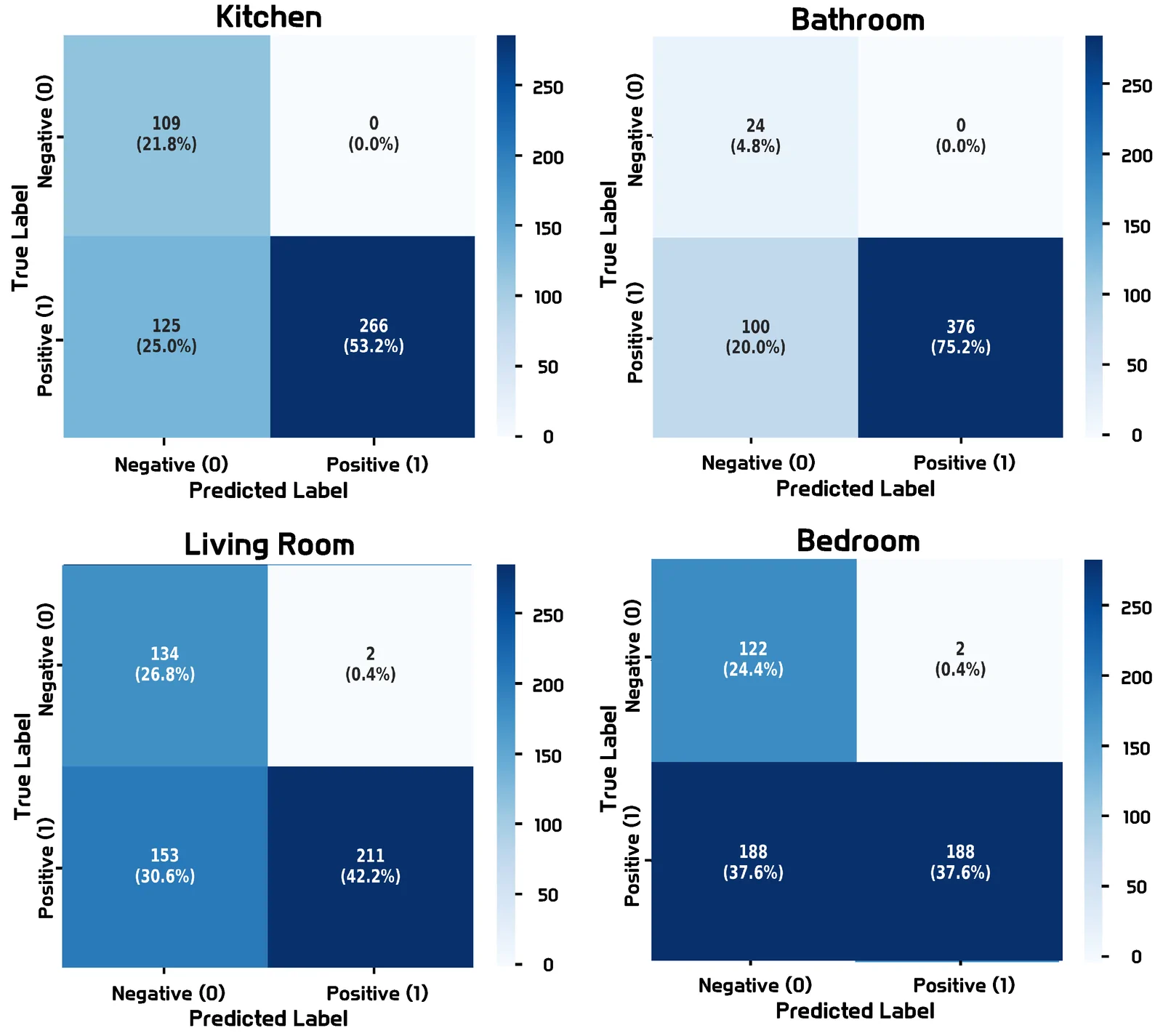
Trajectory assessment confusion matrices across four task domains using the primary VLM evaluator. The matrices highlight the near-zero false-positive behavior of the proposed evaluator, with false-positive rates below 1% in all evaluated environments.

We first analyze the operating behavior and diagnostic characteristics of our primary VLM evaluator (Gemini 1.5 Pro). To quantify the evaluator’s reliability, we map its binarized predictions across tasks using domain-specific 2×2 confusion matrices, as illustrated in Figure 4.

The matrices reveal a highly conservative action-conditioned assessment characteristic across all workspaces. The system consistently achieves a precision approaching 100%, demonstrating that it rarely assigns the maximum score to failed trajectories. Reducing false-positive feedback is important because erroneous success signals may reinforce unsuccessful behavior. Conversely, recall is lower than accuracy and precision because the VLM frequently assigns a score of 2 rather than 3 to successful trajectories when the correspondence between the executed action and the observed visual transition is ambiguous. This conservative behavior reduces false-positive feedback but fails to identify some successful trajectories.

### 6.2. Temporal Window Optimization

**Figure 5:**
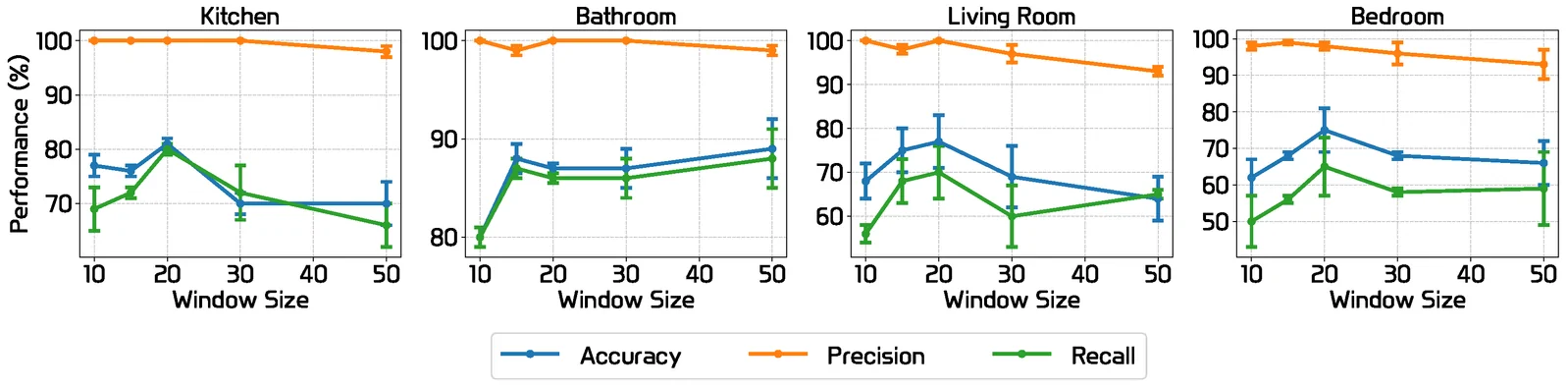
Impact of the temporal window size (segment length) on action-conditioned assessment reliability. The analysis demonstrates a system trade-off between contextual fragmentation (narrow windows) and visual input downsampling and increased inference cost (wide windows).

We examine how the temporal segment length affects trajectory-assessment reliability. Figure 5 tracks the performance metrics (accuracy, precision, and recall) across different temporal window sizes.

The results indicate a clear system trade-off regarding the temporal resolution. If the segment length is too narrow, the temporal context is broken, hindering the model’s ability to reason about action-conditioned visual transitions. Conversely, extending the window size beyond 20 frames causes a degradation in performance due to visual input downsampling and increased reasoning difficulty for large composite prompts. A segment length of 20 achieves the strongest diagnostic reliability in the offline assessment analysis. However, we use a segment length of 10 in the closed-loop control experiments to reduce inference cost while maintaining competitive assessment performance.

### 6.3. VLM Backbone Benchmarking and Latency Trade-Offs

For practical closed-loop control applications, system latency is as critical as accuracy. We benchmark various VLM backbones, categorizing them into larger VLMs (Gemini 1.5 Pro, GPT-4o, Qwen2-VL-72B) and lightweight VLMs (Gemini 1.5 Flash, GPT-4o mini).

**Figure 6:**
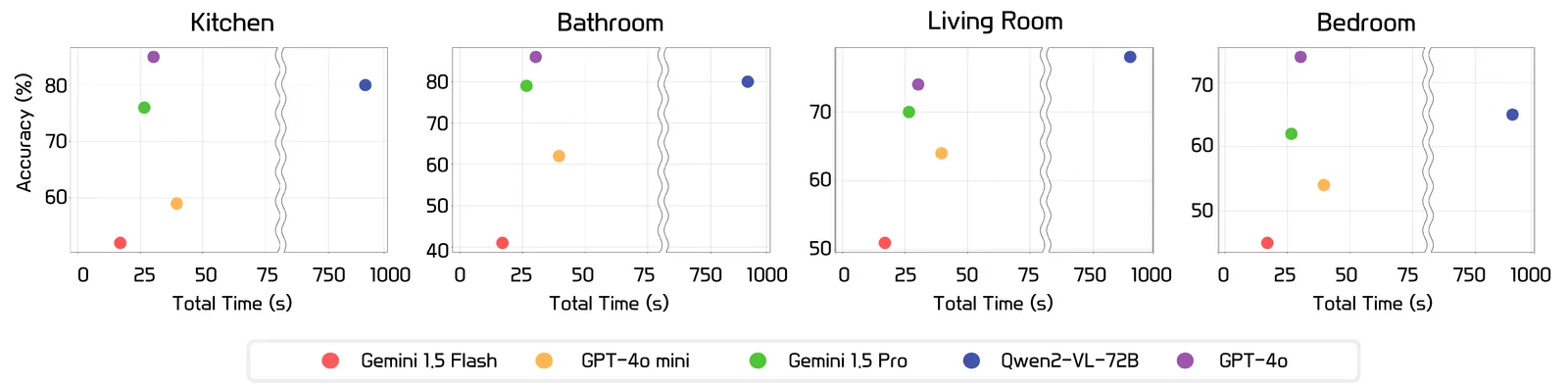
System feasibility trade-off analysis mapping end-to-end computational latency against overall evaluation reliability. Larger VLMs generally occupy the higher-accuracy, higher-latency region, whereas lightweight VLMs provide lower-latency but less reliable assessment.

Figure 6 illustrates the trade-offs between total execution latency and evaluation accuracy. Lightweight VLMs have lower latency but exhibit lower accuracy and precision. Flagship-tier models provide higher reliability but require longer inference times. Crucially, our system latency profiling reveals that over 90% of the end-to-end processing delay occurs during the Stage 1 (Action-Induced Transition Summarization) phase (see Table A2 in Appendix A for the per-stage breakdown), whereas the Stage 2 (Task-Progress Assessment), which processes compressed semantic text, contributes negligibly to the overall overhead. These measurements show that Stage 1 is the principal computational bottleneck. Gemini 1.5 Pro provides a favorable accuracy-latency trade-off among the evaluated backbones, but its approximately 25-s Stage 1 latency remains unsuitable for high-frequency real-time control.

### 6.4. Assessment-Driven Policy Optimization

**Figure 7:**
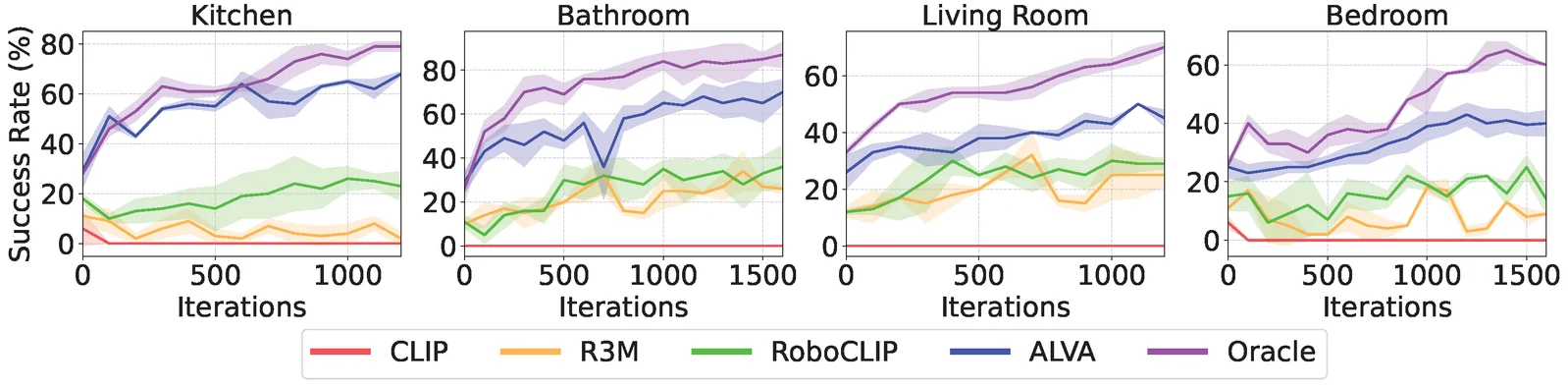
Closed-loop autonomous control performance across four simulated 3D embodied workspaces. The curves track the task completion rate over continuous control optimization iterations for Kitchen, Bathroom, Living Room, and Bedroom environments. The solid lines denote the mean performance, while the shaded regions indicate the standard deviation.

We next evaluate whether the trajectory-level scores can support downstream policy optimization. Figure 7 shows task completion rates over optimization iterations.

To summarize the best observed policy performance during optimization, Table 1 reports the maximum task-completion rate achieved by each method.

In the evaluated simulated tasks, ALVA achieves higher maximum task-completion rates than CLIP, R3M, and RoboCLIP and reduces the gap to the ground-truth oracle. These results are limited to the evaluated zero-shot visual and video-language baselines under the shared observation-based control pipeline.

### 6.5. Ablation Study on Component Configurations and Feedback Types

To understand the architectural drivers behind the success of the ALVA framework demonstrated above, we conduct an ablation study. We decompose this analysis into two parts: evaluating the impact of data fusion configurations (Table 2), and comparing structural feedback paradigms (Table 3).

The video-only configuration provides lower evaluation reliability than the configurations that include executed actions. Adding the action sequence produces the largest improvement among the evaluated structural inputs, indicating that action context helps the VLM relate visual changes to the agent’s executed commands.

Table 3 shows that evaluative scoring yields higher diagnostic metrics than preference-based feedback under the fully integrated input configuration. In this experiment, the discrete rating provides an absolute estimate of task progress, whereas pairwise preference feedback does not directly encode the degree of completion.

## 7. Discussion

The experiments show that action- and language-conditioned video assessment can provide useful trajectory-level feedback for the evaluated simulated household tasks. Compared with final-frame image-text matching, ALVA first interprets visual transitions in the context of the executed action sequence and then estimates progress toward the instruction. The ablation results indicate that both action context and temporal annotations improve assessment reliability. However, these findings are limited to the evaluated environments, tasks, prompts, and VLM backbones.

### 7.1. Limitations

The conservative scoring behavior of ALVA reduces false-positive feedback at the cost of lower recall, as some successful trajectories receive scores below the maximum. Although closed-loop optimization does not use the binary score, under-scoring successful trajectories may weaken the learning signal and slow policy improvement.

Assessment reliability also depends on the VLM backbone, prompt formulation, and API behavior. Prompt-paraphrase sensitivity and repeated-query stability were not evaluated, and the experiments do not include a direct one-stage VLM success-judgment baseline. Table 2 provides aggregate evidence for the value of action logs, but paired qualitative comparisons with and without action logs are not included.

The current implementation is suited to episodic or segment-level assessment rather than high-frequency control. Stage 1 accounts for more than 90% of the measured latency. Moreover, the controlled visibility setting simplifies visual interpretation, and the evaluation is restricted to simulated ALFRED and AI2-THOR environments. Consequently, robustness to natural occlusion and real-world deployment remains unverified. Finally, all methods use replay buffers initialized with human-collected demonstrations; demonstration-free learning from scratch was not evaluated.

### 7.2. Future Work

Future work should evaluate the framework under natural occlusion, imperfect action execution, and observations collected from physical robots. It should also include a direct one-stage VLM success-judgment baseline, test prompt-paraphrase and repeated-query stability, and examine demonstration-free learning. Efficiency improvements should target Stage 1 through sparse frame selection, distilled or fine-tuned transition summarizers, and reusable visual-context caches. Richer outputs could identify failed sub-steps or the observation segments that most influenced the final score.

## 8. Conclusions

We presented ALVA, an action- and language-conditioned video assessment framework for trajectory-level task-progress feedback. The framework summarizes visual transitions conditioned on executed actions and then evaluates the summary with respect to a natural-language instruction. When used as terminal feedback in the evaluated simulated household tasks, ALVA achieved higher closed-loop task-completion rates than the tested static-image and embedding-similarity baselines and narrowed the gap to the ground-truth oracle. These findings are limited to the tested environments, VLM backbones, and observation-based baselines. The current implementation remains limited by conservative scoring, dependence on VLM selection and prompt formulation, controlled object visibility, and substantial Stage 1 latency.

## Figures and Tables

**Table 1:** Maximum task completion rates achieved across different evaluation-driven control models within the four simulated workspaces. Bold indicates the best performance among the non-oracle methods.

Model	Kitchen	Bathroom	Living Room	Bedroom	Average
Ground-Truth Oracle	79.0 ± 2.0	87.0 ± 6.0	70.0 ± 2.0	65.0 ± 3.0	75.3 ± 3.3
CLIP [4]	6.0 ± 7.0	0.0 ± 0.0	0.0 ± 0.0	6.0 ± 2.0	3.0 ± 2.3
R3M [7]	11.0 ± 3.0	34.0 ± 9.0	32.0 ± 8.0	18.0 ± 2.0	23.8 ± 5.5
RoboCLIP [9]	26.0 ± 5.0	36.0 ± 10.0	30.0 ± 5.0	25.0 ± 4.0	29.3 ± 6.0
ALVA (Ours)	**68.0 ± 1.0**	**70.0 ± 6.0**	**50.0 ± 0.0**	**43.0 ± 4.0**	**57.8 ± 2.8**

**Table 2:** Ablation analysis tracking evaluation reliability across different data fusion configurations. All configurations utilize Evaluative scoring to isolate the impact of ‘Temporal’ (timestep annotations) and ‘Control’ (executed action logs) inputs. F1 is averaged across environments and therefore need not equal the harmonic mean of the reported mean precision and recall. Bold indicates the best performance among methods.

Configuration	Acc. (%)	Prec. (%)	Rec. (%)	F1 (%)
Video Only	45.3 ± 3.3	92.3 ± 2.3	35.5 ± 2.8	50.8 ± 3.9
+ Temporal	48.3 ± 3.5	97.3 ± 1.3	38.3 ± 3.8	54.5 ± 4.4
+ Control	64.8 ± 4.0	99.3 ± 0.5	56.0 ± 3.8	70.9 ± 3.4
+ Temporal + Control (Ours)	**71.5 ± 3.5**	**99.5 ± 0.8**	**63.8 ± 3.8**	**77.2 ± 3.4**

**Table 3:** Performance comparison between preference-based and evaluative feedback paradigms. Both models utilize the fully integrated data stream. Bold indicates the best performance among methods.

Feedback Type	Acc. (%)	Prec. (%)	Rec. (%)	F1 (%)
Preference-based	38.3 ± 5.5	88.8 ± 4.3	27.5 ± 6.3	41.8 ± 4.7
Evaluative (Ours)	**71.5 ± 3.5**	**99.5 ± 0.8**	**63.8 ± 3.8**	**77.2 ± 3.4**

## Data Availability

The data used in this study are based on publicly available embodied instruction-following environments and benchmarks. The ALFRED benchmark is available at https://askforalfred.com (accessed on 3 August 2026). Additional implementation details, prompts, and experimental results are available from the corresponding author upon reasonable request.

## Abbreviations

    The following abbreviations are used in this manuscript:

AI2-THOR	Allen Institute for Artificial Intelligence The House Of inteRactions
ALVA	Action- and Language-Conditioned Video Assessment
CLIP	Contrastive Language-Image Pre-training
IQL	Implicit Q-Learning
R3M	Reusable Representation for Robotic Manipulation
RGB	Red-Green-Blue
VLM	Vision-Language Model

## Appendix A. Implementation and Environment Details

### Appendix A.1. Downstream Policy Optimization Details

Although the main focus of ALVA is action- and language-conditioned video assessment and trajectory-level task assessment, we use an online off-policy control module to evaluate whether the generated feedback can support autonomous task execution. Specifically, we use IQL [51] as the downstream policy optimization algorithm, following prior work on reinforcement learning in ALFRED-like embodied environments [3,52]. The hyperparameters used in our experiments are summarized in Table A1.

**Table A1:** Hyperparameters used for downstream off-policy policy optimization.

Parameter	Value
Batch size	128
Training steps	800k for Bedroom, 500k otherwise
Learning rate	$1\times 10^{-4}$
Optimizer	AdamW
Dropout rate	0.1
Weight decay	0.1
Discount factor γ	0.97
Q update Polyak averaging coefficient	0.005
Policy and Q update period	8 per training iteration
Advantage clipping	[0,100]
IQL inverse temperature β	5
IQL expectile parameter τ	0.5
Maximum context length	8

During training, the buffer is initialized with a small number of human-collected demonstrations to reduce exploration time in the long-horizon embodied environments, following the initialization protocol used in prior ALFRED-based policy learning settings [52]. These demonstrations are inserted into the replay buffer only; the policy is not pretrained through behavioral cloning or another imitation-learning objective. All compared methods use the same initialization protocol. The experiments therefore do not evaluate demonstration-free learning from scratch. For ALVA, the progress feedback scores generated by the vision-language evaluator are stored with the corresponding trajectories and used as trajectory-level feedback for downstream off-policy optimization.

### Appendix A.2. Embodied Environment Details

We evaluate ALVA using a modified version of the ALFRED benchmark [3], which provides visually grounded household environments and natural language instructions for embodied task execution. ALFRED is built on the AI2-THOR simulator [13] and was originally designed for imitation learning. We use a modified version that supports reinforcement-learning-based interaction through a gym-style interface [52].

We further modify the environment to improve the visibility of manipulated objects. In the original setup, when the agent picks up certain objects, the object may be partially or fully occluded by the agent’s view, especially for larger objects. Since ALVA evaluates trajectories from visual observations, we ensure that picked-up objects remain clearly visible in the agent’s view. This controlled visibility setting is applied to every evaluated method. It supports consistent comparison under observable object states, but the present experiments do not evaluate robustness to natural occlusion or real-world visual ambiguity.

We define evaluation tasks by randomly sampling 10 tasks from each of four unseen ALFRED floor plans, corresponding to Kitchen, Bedroom, Living Room, and Bathroom environments. This results in 40 tasks in total. Each task is constrained to contain two sub-tasks. For tasks originally containing more than two sub-tasks, only the first two sub-tasks are used. An episode is considered successful only when both sub-tasks are completed.

The agent receives RGB visual observations with a resolution of 224×224. For downstream policy optimization and baseline methods, each observation is encoded using a frozen ResNet-18 pre-trained on ImageNet [53], resulting in a 512×7×7 visual feature representation. The ALFRED action space consists of five navigation actions: MoveAhead, RotateRight, RotateLeft, LookUp, and LookDown; and seven interaction actions: Put, Pickup, Open, Close, ToggleOn, ToggleOff, and Slice [3]. For interaction actions, the policy additionally predicts 1 of 82 object types. Due to the large discrete action space, invalid action–object combinations are masked during policy optimization, following prior work [52]. For the ALVA video interpretation prompt, we use the executed action names but do not include object-type predictions.

Table A2 reports the per-stage querying latency measured for each VLM backbone, complementing the latency–reliability trade-off shown in Figure 6.

**Table A2:** Per-stage querying latency for the benchmarked VLM backbones. Stage 1 (Action-Induced Transition Summarization) dominates the end-to-end cost across all backbones, whereas Stage 2 (Task-Progress Assessment) operates on compressed text and remains negligible.

VLM	Stage 1 (s)	Stage 2 (s)	Total (s)	Stage 1 Share (%)
Gemini 1.5 Flash	15.7	1.3	17.0	92.4
GPT-4o mini	38.8	0.8	39.6	98.0
Gemini 1.5 Pro	25.0	1.6	26.6	94.0
Qwen2-VL-72B	912.2	3.6	915.8	99.6
GPT-4o	29.5	0.8	30.3	97.4

## Appendix B. Evaluation Task Lists

Table A3, Table A4, Table A5 and Table A6 list the task instructions used in the four embodied environments. These instructions are included to clarify the language-conditioned tasks used for trajectory-level evaluation and to support reproducibility.

**Table A3:** Task instructions used in the Kitchen environment.

Task No.	Sub-Task Type	Instruction
1	PickupObject	Pick up the spoon from the counter.
	PutObject	Put the spoon in the white cup on the shelf.
2	PickupObject	Pick up the egg that is beside the fork in the sink.
	CoolObject	Open the refrigerator, then place the egg on the glass shelf and close the fridge. Wait, then open the fridge and pick up the egg, then close the fridge.
3	PickupObject	Pick up the tomato from the sink.
	CoolObject	Open the fridge door, put the tomato inside of the fridge, close the door, open the door, take the tomato out, close the door.
4	PickupObject	Pick up the mug in the coffee maker.
	CoolObject	Open the fridge, put the cup in the fridge, close the fridge, wait, open the fridge, pick the cup, close the fridge.
5	PickupObject	Pick up the bread.
	CoolObject	Open the fridge, put the bread in the fridge, close the fridge, open the fridge, get the bread, and close the fridge.
6	PickupObject	Pick up the white coffee cup to the right of the trophy.
	CleanObject	Put the coffee cup in the sink, turn on the water, turn off the water, and pick up the coffee cup.
7	PickupObject	Pick up the smaller silver knife on the counter.
	PutObject	Put the knife in the green cup in the sink.
8	PickupObject	Pick up a bowl from the shelf.
	PutObject	Put the bowl on the counter.
9	PickupObject	Grab the knife from the counter.
	PutObject	Put the knife in the pan on the stove.
10	PickupObject	Pick up the knife from the counter.
	CleanObject	Place the knife in the sink and turn the water on. Turn the water off and pick up the knife.

**Table A4:** Task instructions used in the Bedroom environment.

Task No.	Sub-Task Type	Instruction
1	PickupObject	Pick up the bowl from the shelf.
	ToggleObject	Turn on the lamp sitting on the desk while holding the bowl.
2	PickupObject	Pick up the white mug from the desk.
	ToggleObject	Turn the desk lamp on with the mug in hand.
3	PickupObject	Pick up the book from the bed.
	ToggleObject	Turn on the lamp on the desk while carrying the book.
4	PickupObject	Pick up the mug from the shelf.
	ToggleObject	Turn the lamp on while holding the cup.
5	PickupObject	Pick up the bowl on the desk.
	ToggleObject	Turn on the lamp on the desk while holding the bowl.
6	PickupObject	Pick up the pencil from the desk.
	PutObject	Put the pencil in the bowl.
7	PickupObject	Pick up the alarm clock from the desk.
	ToggleObject	Turn on the lamp on the desk while holding the alarm clock.
8	PickupObject	Pick up the clock from the back of the desk.
	ToggleObject	Hold the clock and turn on the lamp on the right side of the desk.
9	PickupObject	Pick up the mug on the shelf.
	PutObject	Put the mug on the desk.
10	PickupObject	Pick up the pencil on the desk.
	PutObject	Place the pencil in the glass bowl on the desk.

**Table A5:** Task instructions used in the Living Room environment.

Task No.	Sub-Task Type	Instruction
1	PickupObject	Pick up the cell phone from the dresser.
	ToggleObject	Hold the cell phone and turn the lamp on.
2	PickupObject	Pick up the remote that is on the blue chair.
	ToggleObject	Turn on the lamp with the remote in hand.
3	PickupObject	Pick up the laptop on the right after closing it.
	ToggleObject	Turn on the floor lamp while carrying the laptop.
4	PickupObject	Pick the phone up from the desk.
	ToggleObject	Turn the lamp on while holding the phone.
5	PickupObject	Grab the tissue paper from the dresser.
	ToggleObject	Carry the tissue as you turn on the lamp.
6	PickupObject	Pick up the remote from the middle of the dresser, directly behind the tissues.
	ToggleObject	Hold the remote and turn on the lamp.
7	PickupObject	Pick up a pillow from the chair.
	PutObject	Put the pillow on the couch.
8	PickupObject	Pick up the statue on the top shelf.
	ToggleObject	Turn on the lamp while holding the statue.
9	PickupObject	Pick up a statue from the dresser.
	ToggleObject	Turn on the floor lamp with the statue in hand.
10	PickupObject	Pick up the left pillow on the chair.
	PutObject	Put the pillow on the sofa right of the newspaper.

**Table A6:** Task instructions used in the Bathroom environment.

Task No.	Sub-Task Type	Instruction
1	PickupObject	Pick up the bar of soap on the back of the toilet.
	PutObject	Place the soap in the trash can.
2	PickupObject	Pick up bar of soap.
	CleanObject	Put soap in sink, turn water on, turn water off, remove soap from sink.
3	PickupObject	Pick up the cloth from the counter.
	CleanObject	Put the cloth in the sink and turn the water on and then off and pick the cloth up from the sink.
4	PickupObject	Pick the soap up from the back of the toilet.
	CleanObject	Put the soap in the sink and turn the water on and then off and pick up the soap again.
5	PickupObject	Pick the cloth up from the counter.
	CleanObject	Put the cloth in the sink and turn the water on and then off and take the cloth out of the sink.
6	PickupObject	Pick up the bar of soap.
	CleanObject	Put the bar of soap in the sink, turn the water on and then off and then pick up the bar of soap.
7	PickupObject	Pick up the bar of soap on the back of the toilet.
	PutObject	Open the cabinet, put the bar of soap inside, and close the cabinet.
8	PickupObject	Grab a bar of soap off of the counter.
	PutObject	Put the soap in the trash can.
9	PickupObject	Pick up the soap on the counter.
	PutObject	Open the cabinet and put in the soap then close the cabinet.
10	PickupObject	Pick up toilet roll from off the toilet.
	PutObject	Open sink cabinet and place roll inside before closing the door.
